# Screening of Serum Exosomal miRNAs as Diagnostic Biomarkers for Gastric Cancer Using Small RNA Sequencing

**DOI:** 10.1155/2022/5346563

**Published:** 2022-05-04

**Authors:** Jun-fu Wang, Yue-mei Jiang, Wen-hui Zhan, Shan-ping Ye, Tai-yuan Li, Jiang-nan Zhang

**Affiliations:** ^1^Department of General Surgery, The First Affiliated Hospital of Nanchang University, Nanchang 330031, China; ^2^Department of Gastrointestinal Surgery, The First Affiliated Hospital of Guangxi Medical University, Nanning 530021, China; ^3^Department of Prosthodontics, The Affiliated Stomatological Hospital of Jiujiang University, Jiujiang, Jiangxi 332000, China; ^4^Department of Maxillofacial Surgery, The Affiliated Stomatological Hospital of Jiujiang University, Jiujiang, Jiangxi 332000, China

## Abstract

**Background/Aim:**

Exosomal miRNAs are promising tumor biomarkers. This research explored the diagnostic value of serum exosomal miRNAs by analyzing the exosomal miRNAs derived from the serum of gastric cancer patients.

**Methods:**

Deep sequencing of exosomal miRNAs was performed using an Illumina HiSeq2500 sequencer on serum samples from three healthy subjects in the normal control group (group N) and six gastric cancer patients in the gastric cancer treatment group (group T). Bioinformatics analysis was performed on exosomal miRNA profiles to screen differentially expressed miRNA. In addition, target gene prediction, GO, and KEGG pathway enrichment analyses were performed. Finally, the serum exocrine bodies of 24 patients with gastric cancer and 24 normal controls were verified by quantitative reverse transcription-polymerase chain reaction (qRT-PCR) to confirm the findings. The receiver operating characteristic (ROC) curve of the subjects was plotted, and the area under the curve (AUC) was calculated with a 95% confidence interval (CI).

**Results:**

The exosomes were successfully extracted from the serum of gastric cancer patients, which showed a form of goblet vesicles or irregular circles, with an average particle size of approximately 102.3 nm. The exosomal marker proteins, CD9, CD63, TSG101, and calnexin, were positively expressed. Small RNA sequencing detected 15 different types of RNA components in the serum exosomes, and the most abundant one was miRNA. In the screened cohort, the downregulation of seven existing miRNAs and the upregulation of one existing miRNA were observed. Four of them were selected for confirmation, revealing that the expression of miR-10401-3p, miR-1255b-5p, and miR-6736-5p declined significantly in group T (*P* < 0.05). In addition, the ROC curve showed that the AUC values for these three miRNAs were 0.8333, 0.8316, and 0.8142, respectively; all of them are statistically significant (*P* < 0.05).

**Conclusions:**

The above three miRNAs found in the serum exosomes from gastric cancer patients might serve as diagnostic biomarkers for gastric cancer.

## 1. Introduction

Gastric cancer is a common malignant tumor in the digestive system, ranking second in morbidity and third in mortality among all tumors in China [1], and represents a serious threat to human health. Currently, pathological biopsy during gastroscopy is the main diagnostic approach for gastric cancer. However, it is not a conventional screening method because of its invasiveness and the lack of sensitive and specific indices for early screening and diagnosis. Therefore, it is of paramount importance to find noninvasive, sensitive, and highly specific biomarkers.

miRNAs are highly conserved, single-stranded RNAs of approximately 18–24 nucleotides in length that play a critical role in the posttranscriptional regulation of gene transcription [2, 3]. The abnormal expression of miRNAs has been confirmed in multiple tumors, with an important role in the development and progression of tumors [4, 5]. Many studies identified several valuable miRNAs for the screening, diagnosis, and treatment of gastric cancer [6, 7]. A growing number of studies suggest that miRNAs are secreted by extracellular vesicles (exosomes, vesicles, and apoptotic bodies), affecting the biological processes of the recipient cells through their intercellular interactions [8].

Exosomes are bioactive, cup mouth-shaped, and lipid bilayer-structured nanoscale vesicles actively secreted by cells, with a diameter of approximately 40–100 nm [9–11]. They have cell-derived membrane molecules on their surface and are rich in several protein types, lipids, mRNAs, miRNAs, double-stranded DNAs, and other important substances [12–15]. As star molecules in exosomal research, miRNAs benefit from the fact that exosomes protect them from degradation by RNA enzymes, enhancing their stability and role in biological processes [16]. Exosomes are extensively diffused in human body fluids and tissues. Some scientists found that significantly more exosomes are secreted by tumor tissues and cells (at least ten times) than by the normal ones [17, 18]. In addition, exosomes exchange cellular information through the transfer of miRNAs, growth factors, and other molecules and serve as means of transfer and information exchange among cells [19, 20]. Some studies also revealed that exosomal miRNAs mediate signal transduction between tumors and the tumor microenvironment [21, 22]. Nowadays, exosomal miRNAs are a research hotspot, and more and more studies are focusing attention on the role of exosomes in the initiation, progression, and clinical treatment of tumors, including tumor invasion and metastasis, angiogenesis, antitumor immunity, and tumor immune escape, thanks to the technological advances [23, 24]. Liu and Chu found that exosomal miRNAs as circulating biomarkers could predict the development of hematogenous metastasis after surgery for stage II/III gastric cancer [7].

Therefore, this study analyzed the expression of serum exosomal miRNAs from healthy subjects and gastric cancer patients using small RNA sequencing. The results of the two groups were compared by the bioinformatics analysis to evaluate the statistical difference. In addition, quantitative reverse transcription-polymerase chain reaction (qRT-PCR) was used to confirm the results of the selected differentially expressed miRNAs that could be thus considered biomarkers with potential diagnostic values.

## 2. Materials and Methods

### 2.1. Clinical Data and Sample Collection and Preparation

Serum samples were collected from gastric cancer patients admitted into the Department of Gastrointestinal Gland Surgery, the First Affiliated Hospital of Guangxi Medical University, China, between January and December 2019. All the patients were diagnosed with gastric cancer by electronic gastroscopic biopsy. Other systemic tumors, tumors metastasizing to the stomach, and other basic diseases were excluded. The patients did not receive chemotherapy or radiotherapy before the operation. Healthy volunteers provided normal healthy serum samples. In the early stage, three normal healthy subjects and six gastric cancer patients' sera were collected for high-throughput sequencing. In the later period, 24 cases of gastric cancer patients and 24 cases of normal healthy serum were collected for qRT-PCR verification. This study was approved by the Ethics Committee of the First Affiliated Hospital of Guangxi Medical University, and all patients signed written informed consent forms. A total of 15 mL of blood was collected from each patient and placed in a still-standing state for 15 min before centrifugation at 3,000 × *g* for 15 min at 4°C. The supernatant (blood serum) was collected, placed in a new tube, and centrifuged at 12,000 × *g* for 30 min at 4°C to remove cellular fractions. The separated serum samples were stored at −80°C for further use.

### 2.2. Separation and Purification of Exosomes

Exosomes were separated using an exoRNeasy Serum Starter Kit (Qiagen GmbH, Hilden, Germany). 5 mL of serum was mixed with 5 mL of XBP buffer in a test tube, which was then gently inverted five times. The serum/XBP mixture was added to an exoEasy column spinner and centrifuged at 500 × *g* for 1 min. The waste supernatant was discarded, and the column was placed back into the collection tube. Next, 10 mL of XWP buffer was added to an exoEasy Midi column spinner, which was washed by rotation at 5,000 × *g* for 5 min. The waste supernatant and the collection tube were discarded, and the column spinner was transferred to a new collection tube. Finally, an appropriate amount of PBS was added to the column spinner, and exosomes were collected after 5 min of centrifugation at 5,000 × *g*, transferred to a 1.5 mL centrifuge tube, and stored at −80°C for further use.

### 2.3. Nanoparticle Tracking Analysis (NTA)

The exosomes were diluted in PBS (1 : 1,000) and thoroughly mixed with single particles. The diluted exosomes were injected into a ZetaView NTA (avoiding the presence of bubbles during injection) by a syringe to observe the Brownian motion of each nanoparticle, and the size and concentration of exosomes were calculated.

### 2.4. Transmission Electron Microscopy (TEM)

The sample rich in exosomes was dissolved in 0.01 mol/L PBS, and 10 *μ*L of the suspension was dripped onto a copper mesh, followed by 5 min of still standing. The copper mesh was dried using filter paper for several minutes at room temperature. A drop of 2% uranyl acetate was dripped on it, and the copper mesh was again dried using filter paper. Finally, TEM at 80 KeV was used for observation.

### 2.5. Western Blot

The exosome sample was lysed using RIPA lysis buffer, and the protein concentration was determined by the BCA method. Next, 30 *μ*g of protein was collected from each sample, subjected to electrophoretic separation, and transferred to a PVDF membrane. The PVDF membrane was sealed with 5% skim milk at room temperature for 30 min, and then specific antibodies (CD9, CD63, TSG101, and calnexin) were added and incubated overnight at 4°C. Next, the PVDF membrane was washed using TBST for 10 min (three times), and horseradish peroxidase (HRP)-conjugated anti-rabbit/mouse IgG was added and incubated at room temperature for 1 h. Finally, the membrane was washed using TBST for 10 min (three times) and observed under a fluorescence detection system by chemiluminescent immunoassay.

### 2.6. Extraction of Exosomal miRNAs

A total of 700 *μ*L of QIAzol lysis buffer was added to the exosome samples and incubated at room temperature for 5 min. Next, 90 *μ*L of chloroform was added, followed in succession by 15 s of shaking, 2–3 min of incubation at room temperature, and 15 min of centrifugation at 12,000 × *g*and 4°C. The supernatant was transferred into a new collection tube, and anhydrous ethanol was added at an amount 1.5 times the total volume and thoroughly mixed using a straw. Subsequently, 700 *μ*L of the mixture (including any sediment) was collected with a straw and added to the RNeasy MinElute spin column (already transferred into a 2-mL collection tube). The tube was centrifuged at 8,000 × *g* at room temperature for 15 s, and the supernatant was discarded. The above step was repeated until the mixture was entirely filtered. Next, 700 *μ*L of RWT buffer was added to the RNeasy MinElute spin column, followed by centrifugation at 8,000 × *g*for 15 s three times. The waste supernatant was discarded, and the RNeasy MinElute spin column was placed into a new centrifuge tube for full-speed centrifugation for 1 min to dry the membrane. Next, the RNeasy MinElute spin column was transferred into a new 1.5-mL centrifuge tube, and 14 *μ*L of enzyme-free water was added and absorbed onto the spin column membrane. After 1 min of capped centrifugation at 8,000 × *g*, filtration was performed to obtain exosomal total RNA for further use.

### 2.7. miRNA Library Construction and Deep Sequencing

Exosomal total RNA was used for the construction of the miRNA library and sequencing by Gene Denovo Biotechnology Co., Ltd. (Guangzhou, China). The extracted total RNA sample was subjected to agarose gel electrophoresis, and 18-30-nt fragments were selected. Next, 3′ adapters were connected to enrich 36-44-nt RNAs, and 5′ adapters were connected to RNAs. The following step was taken to perform the reverse transcription of PCR amplification and ligate products, amplify the 140-160-bp PCR products, and construct a cDNA library. The constructed library was subjected to quality control using Agilent 2100 and qPCR, and small RNA deep sequencing was performed using the Illumina HiSeq TM2500 platform.

### 2.8. RNA-Seq Data Analysis

**Figure 1:**
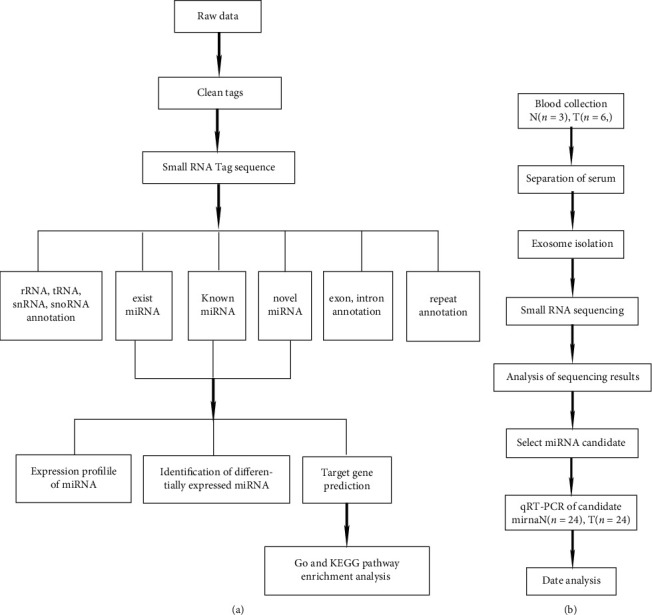
Bioinformatics analysis and flowchart of experiments. (a) Flowchart of bioinformatics analysis and (b) flowchart of experiments.

The small RNA data obtained through the preliminary filtration of the raw reads were further filtered to remove low-quality reads and acquire the high-quality ones. After eliminating the adapters and filtering out small RNA tags with a read frequency number of <2, a small RNA clean tag sequence was eventually obtained for subsequent analysis. The small RNAs were compared with the RNAs in the GenBank database (Release 209.0), Rfam database (Release 11.0), and genomes to identify existing miRNAs, known miRNAs, and novel miRNAs. According to this procedure, the small RNAs were annotated by types, the existing and known miRNAs were identified according to the miRbase database (Release 22), and novel miRNAs were identified according to Mireap v 0.2 software. Next, the miRNAs identified with each sample were summarized, the tag per million expression of each miRNA was calculated, and the miRNA expression profile of all the samples was obtained. Then, the cluster heat-map analysis on the expression patterns of miRNAs was performed by analyzing the principal miRNA components, and miRNAs with a tag per million of <1 were filtered out. Finally, the miRNA expression profiles of the two groups were compared, differential analysis on miRNAs was performed using edgeR software, and the significant and differentially expressed miRNAs were identified with log2 (fc) >1.0 and *P* < 0.05 (Figure 1(a)).

### 2.9. Target Gene Prediction of miRNAs and Function Enrichment Analysis on Target Genes

According to the sequences of existing miRNA, known miRNAs, and novel miRNAs, candidate target genes were predicted using the software programs TargetScan 7.0, RNA hybrid 2.1.2 +Svg-light 6.01, and Miranda 3.3a. The intersections among the results predicted by the three software programs represented the target genes of the miRNAs. A miRNA-mRNA targeted relationship network was also constructed using the Cytoscape software. DAVID Bioinformatics Website was used to perform GO and KEGG enrichment analysis on the target genes of the differentially expressed miRNAs. A *P* value of <0.05 was considered the condition to screen cancer-related, significantly enriched signal transduction pathways, and the enrichment classes and enrichment scores of all the biological processes, cellular components, and molecular functions, in which differentially expressed miRNAs might be involved, were displayed.

### 2.10. Detection of Exosomal miRNA Expression Using qRT-PCR

The RNA extracted from the exosomes was used for miRNA cDNA synthesis using a PrimeScript RT reagent kit. The expression of miRNAs was detected by qRT-PCR using an SYBR Green I fluorescence detection kit. All the primers used were synthesized by Takara (Table 1). A 7500 Real-Time PCR System was used for quantitative detection, and the relative expression of the miRNAs and U6 in the serum exosomes from healthy subjects and gastric cancer patients was calculated according to the 2^−ΔΔCt^ method. For each sample, three portions were prepared for three independently repeated trials. The results were expressed as mean ± standard deviation.

### 2.11. Data Analysis

Statistical analysis and graphic generation were performed using GraphPad 7.0 software.  Figure 1(b) illustrates the entire trial flow. The paired *t*-test and one-way analysis of variance were used to determine the significance in the relative expression of the miRNAs in the exosomes between or among groups. A *P* value of <0.05 was considered statistically significant. The expression of each miRNA was used to plot the receiver operating characteristic (ROC) curve of the subjects. The area under the curve (AUC) was calculated with a 95% CI, and the accuracy of the ROC analysis system was evaluated for exosomal miRNA identification.

## 3. Results

### 3.1. Separation and Identification of Serum Exosomes

**Figure 2:**
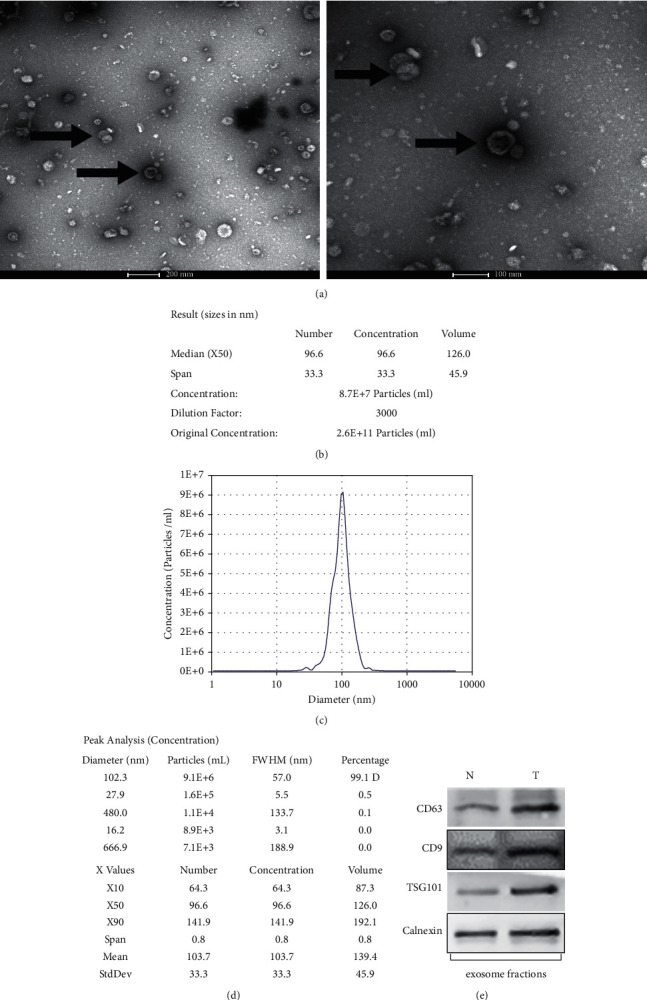
Identification and characteristics of serum exosomes. (a) Representative transmission electron microscopy (TEM) images of exosomes (in the form of goblet vesicles or irregular circles, scale = 100 nm, 200 nm). (b–d) Particle size, concentration, and distribution of exosomes in the NTA report). (e) Expression of exosomal biomarkers CD9, CD63, TSG101, and calnexin detected by western blot.

According to the TEM results, the exosomes possessed the form of goblet vesicles or irregular circles, and their particle size ranged between 30 and 120 nm (Figure 2(a)). In the follow-up detection of exosomes using the ZetaView NTA, the dynamic scattering of these acellular components displayed the particle size and distribution (Figures 2(b)–2(d)). The obtained particles had an average particle size of 102.3 nm and accounted for 99.1% of the total number of particles. The concentration of exosomes in the serum was approximately 2.6*E* + 11 particles/mL; after a 3,000-round dilution procedure, the concentration of the particles was approximately 8.7*E* + 7 particles/mL. Western blot analysis was performed to detect the expressions of the exosomal biomarkers CD9, CD63, TSG101, and calnexin (Figure 2(e)). All the results obtained by TEM, NTA, and western blot confirmed the characteristics of exosomes and proved the successful exosome extraction. The concentration and purity of exosomes also met the requirements for the follow-up miRNA deep sequencing.

## 4. Exosomal miRNA Profiles in Serum

**Figure 3:**
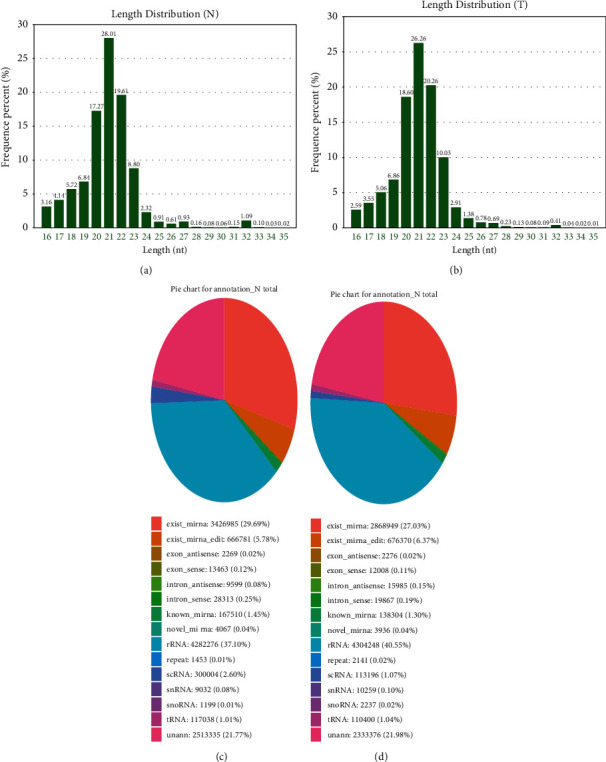
Quality assessment on the sequencing results of serum exosomal miRNA. (a, b) Length distribution of annotated fragments and identified miRNAs in two groups. (c, d) Percentage pie chart of small RNA types in the exosomal miRNA expression profiles of the two groups.

Small RNA sequencing was performed on the serum exosomes from group N (three healthy subjects) and group T (six cases) by Gene Denovo Biotechnology Co., Ltd. using the Illumina HiSeq TM2500 high-throughput sequencer to investigate the miRNA expression of the serum exosomes from gastric cancer patients. Through filtering out the reads containing linkers or low-quality bases, a high-quality small RNA tag sequence meeting the relevant requirements was ultimately acquired. The miRNAs obtained through small RNA sequencing ranged between 16 and 35 nt in length and were most significantly characterized by the emergence of the main peak at 21 bp; their frequency percentages were 28.01% and 26.26%, respectively (Figures 3(a)–3(b). These parameters were consistent with the tag length distribution characteristics of animal samples. Through the database comparison and identification, 15 different types of RNA components were found in serum exosomes, and among them, the most abundant was miRNA. Groups N and T roughly accounted for 29.69% and 27.03% of miRNA, respectively. In addition, rRNA, scRNA, snoRNA, snRNA, piRNA, and other RNAs were traced in small amounts (Figures 3(c) and 3(d).

## 5. Intergroup Comparison of the Differentially Expressed Serum Exosomal miRNAs

**Figure 4:**
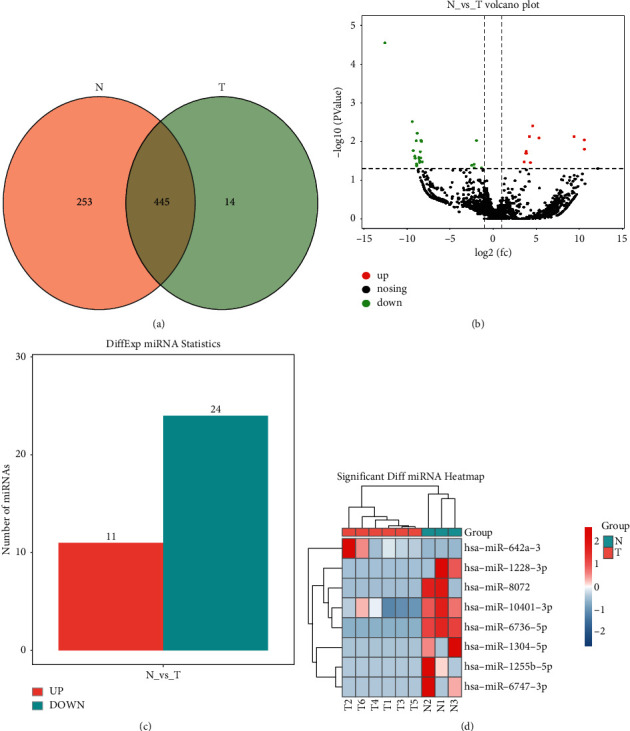
Comparison of differentially expressed miRNAs between groups N and T. (a) Description of shared and specific miRNAs in the two groups using the Venn diagram. (b) Volcano plot of differentially expressed serum exosomal miRNAs in the two groups using edgeR software (red: increase; green: decrease; gray: no difference), log2(fc) >1.0, *P* < 0.05. (c) Statistical analysis on the differentially expressed miRNAs. (d) Upregulated and downregulated miRNAs in the two groups through cluster heat-map analysis.

A total of 698 known miRNAs were identified in group N (including 445 shared miRNAs and 253 specific miRNAs), and 459 known miRNAs were identified in group T (including 445 shared miRNAs and 14 specific miRNAs). Clear differences were found between the two groups in serum exosomal miRNA profiles (Figure 4(a)). Differential analysis was performed on the miRNA-seq data of groups N and T using the edgeR software. The criteria for screening differentially expressed miRNAs were the following: variation in the expression above 1.0 fold and *P* < 0.05.  Figure 4(b) shows the volcano plot of differentially expressed miRNAs identified in the serum exosomes. The software detected 35 significantly dysregulated miRNAs, including 11 upregulated and 24 downregulated ones (see Figure 4(c)). Among them, 8 existing human miRNAs (Table 2), 24 known miRNAs, and 3 new miRNAs were statistically significant. The 8 existing human miRNAs showing differential expression were selected for cluster heat-map analysis (Figure 4(d)), and 7 significantly downregulated miRNAs (miR-10401-3p, miR-1255b-5p, miR-6736-5p, miR-6747-3p, miR-1304-5p, miR-1228-3p, miR-8072) and 1 significantly upregulated miRNA (miR-642a-3p) were found in the T group. These findings suggested that these miRNAs might play a role as diagnostic biomarkers for gastric cancer.

## 6. Target Gene Prediction of Differentially Expressed miRNAs

**Figure 5:**
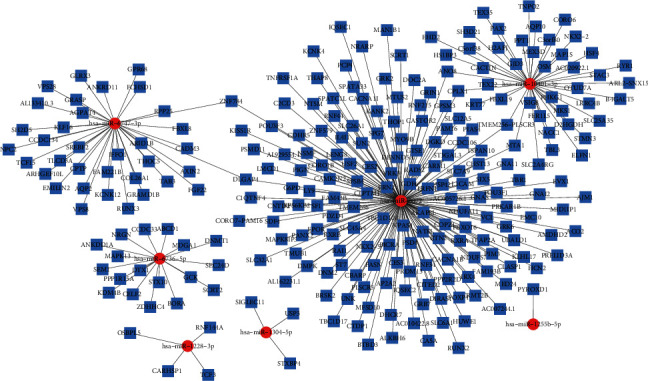
Target gene prediction network of differentially expressed miRNAs. Target gene prediction network of the 8 differentially expressed miRNAs (the potential target genes are marked in blue and miRNAs in red).

The target genes of the 8 screened differentially expressed miRNAs were predicted using 3 databases. The screening condition was as follows: *P* < 0.01 and free energy <35.  Table 3 provides the target gene information on the screened miRNAs.  Figure 5 shows the target gene prediction network of the 8 differentially expressed miRNAs plotted using the Cytoscape software.

## 7. Function Enrichment Analysis on the Target Genes of Differentially Expressed miRNAs

**Figure 6:**
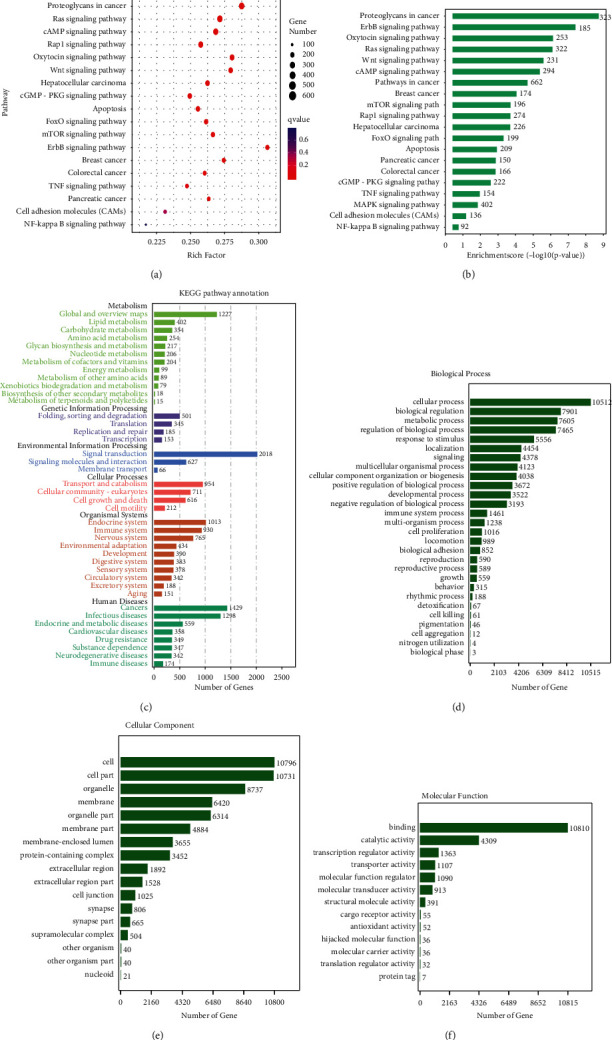
KEGG and GO pathway enrichment analysis. (a) Bubble chart of KEGG enrichment analysis on cancer-related pathways in which the target genes had a role (*P* < 0.05 and *P* < 0.05 after correction). (b) Column chart of the quantity level of the target genes on cancer-related pathways. (c) Distribution of KEGG pathway annotations. (d–f) GO analysis on the enrichment classes of the target genes in biological processes, cellular constituents, and molecular functions.

The target genes of the differentially expressed miRNAs in the two groups were subjected to GO and KEGG pathway enrichment analysis.  Table 4 provides the pathway enrichment results of the target genes of the 8 differentially expressed miRNAs. Adopting *P* < 0.05and *P* < 0.05 after correction as screening conditions, the cancer-related pathways were selected, and the bubble chart of the signaling pathways of the target genes and the column chart of the quantity of the target genes were obtained through the KEGG enrichment analysis (Figures 6(a) and 6(b). The analysis revealed multiple signaling pathways related to tumor proliferation, invasion, metastasis, and apoptosis, such as MAPK, PI3K-Akt, Ras, and Rap1 signaling pathways and other cancer-related pathways. The upregulation or downregulation of miRNAs in the exosomes might cause changes in the target genes of the above signaling pathways, suggesting that these differentially expressed miRNAs play essential roles in cancer. This analysis provided strong evidence for further studies on the mechanism of serum exosomes from gastric cancer patients. The KEGG pathway enrichment analysis also revealed the interaction between and the biological function of the target genes. To determine the function of the differentially expressed miRNAs in the serum exosomes from gastric cancer patients and healthy subjects, the genes showing significantly different expression patterns were identified, GO and KEGG database analysis was performed, and all the genes with different expression patterns were divided into six subgroups, such as metabolism, genetic information processing, environmental information processing, cellular process, organism system, and human diseases. The target gene number of the differentially expressed miRNAs was also listed in the pathway enrichment diagram. A number >1,000 target genes of differentially expressed mRNAs was found, mainly concentrated in the signal transduction (*n* = 2,018) of the environmental information processing subgroup. This confirmed the transmission role of the miRNAs encapsulated in the exosomes in biological processes and their close correlation with tumors. The main human diseases in which these miRNAs had a role were tumors (*n* = 1,429) and infectious diseases (*n* = 1,298), especially tumors. This indicated that the miRNAs encapsulated in the exosomes were closely related to tumors and infectious diseases, as detailed in Figure 6(c). The enrichment classes and enrichment scores of the target genes of the differentially expressed miRNAs were further listed in GO categories such as biological processes, cellular constituents, and molecular functions, as shown in Figure 6(d)–6(f).

### 7.1. Validation of RNA-Seq Data Using RT-PCR

**Figure 7:**
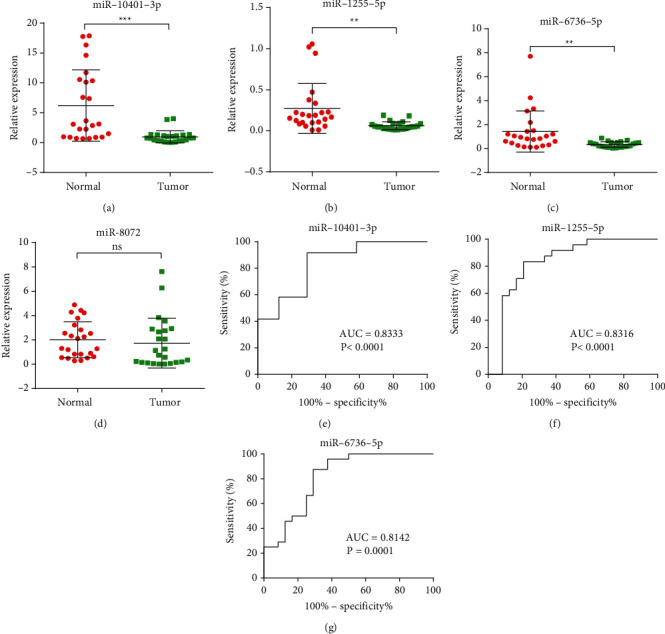
Relative expression and diagnostic value of the candidate exosomal miRNAs using qRT-PCR. (a–d) Confirmation of the relative expression of R-10401-3p, miR-1255b-5p, miR-6736-5p, and miR-8072 in the serum exosomes from group N (*n* = 24) and group T (*n* = 24) using qRT-PCR (U6 used as the internal control, (^*∗∗∗*^*P* < 0.001); (^*∗∗*^*P* < 0.01); (^*∗∗*^*P* < 0.01); (^*∗*^*P* < 0.05). (e) Receiver operating characteristic (ROC) curve of miR-10401-3p, AUC = 0.8333 (*P* < 0.001). (f) ROC curve of miR-1255b-5p, AUC = 0.8316 (*P* < 0.001). (g) ROC curve of miR-6736-5p, AUC = 0.8142 (*P*=0.001).

To evaluate the diagnostic values of serum exosomal miRNAs as biomarkers for gastric cancer, the differential expression of the screened candidate miRNAs was confirmed using qRT-PCR. To be specific, adopting the serum exosome samples from 24 healthy subjects and 24 gastric cancer patients as the object of the study, four downregulated miRNAs were selected for clinical validation, and three biological repetitions were used for each trial. Our results showed that the expression of miR-10401-3p, miR-1255b-5p, and miR-6736-5p significantly decreased in group T compared to group N (*P* < 0.05) (Figures 7(a)–7(c); however, no statistically significant difference was observed between the two groups in the expression of miR-8072 (*P* < 0.05) (Figure 7(d)). The expression of each miRNA was used to plot the ROC curve of the subjects. The AUC with 95% confidence interval (CI) for miR-10401-3p was 0.8333 (*P* < 0.001) (Figure 7(e)); the AUC values for miR-1255b-5p and miR-6736-5p were 0.8316 (*P* < 0.001) and 0.8142 (*P* < 0.001) (Figures 7(f) and 7(g)), respectively. The above results suggested that the miRNAs selected in this study could represent good diagnostic biomarkers for gastric cancer.

## 8. Associations between the Candidate Exosomal miRNAs and Clinical Characteristics in GC Patients

We further evaluated the relationship between the expression of miR in serum exosomes and clinical features in 24 cases of gastric cancer. The results showed that miR-10401-3p was closely related to tumor size (*P*=0.029), miR-1255-5p was closely related to tumor metastasis (*P*=0.004), and miR-6736-5p was closely related to tumor stage (*P*=0.03) and metastasis (*P*=0.045) (Table 5). These results may provide clues for us to study the functional test of miR in serum exocrine in the future.

## 9. Discussion

Exosomes are released in the cells by the multivesicular bodies and plasma membranes after fusion due to specific stimulation or apoptosis. The miRNAs in exosomes are internalized by adjacent or remote cells and regulate the target genes in cells at a posttranscriptional level, affecting the biological functions of recipient cells [25]. Exosomal miRNAs are bridges for cell-cell and cell-environment communication and offer a mechanism used by cells to communicate with each other [26]. Exosomes are detected in almost all the body fluids of the human body, such as blood, urine, ascites, and amniotic fluid. Exosomes have a nuclease hydrolysis membrane, which protects miRNAs from degradation and stabilizes them. These features not only contribute to the progression of research but also turn exosomal miRNAs into convenient, reliable, and stable molecular biomarkers [16]. In recent years, exosomal miRNAs have aroused great interest among scientists in China and other countries, revealing their potential as novel diagnostic biomarkers for tumors.

miRNAs are conserved, noncoding RNAs. A continuous increase in the number of studies on the role of miRNAs in tumors has been observed in the past few years. The expression of miRNAs can be tissue-specific or cell-specific. Their expression can reflect the potential pathophysiologic status. miRNAs are the main functional molecules with a role in intercellular communication when inside exosomes, and the role of exosomal miRNAs in tumors is also attracting increasing attention. Exosomal miRNAs have some potential advantages in serving as diagnostic biomarkers. They can selectively carry and encapsulate miRNA “goods,” and in a separation cycle, exosomal miRNAs can reduce or eliminate interferences from nonexosomal miRNAs to the cycle, which enhances the credibility and reliability of exosomal miRNA profiles in the diagnosis. Over the past few years, several results on miRNA sequencing of serum or plasma exosomes were reported in colorectal [27], lung [28–30], liver [31, 32], pancreatic [33], and thyroid cancers [34], indicating a new approach for tumor diagnosis. The expression of miR-6803-5p in the serum exosomes from colorectal cancer patients is higher than that in the serum exosomes from healthy controls, and its expression in the exosomes is correlated with poor prognosis of colorectal cancer patients (ROCAUC = 0.7399), suggesting that exosomal miR-6803-5p can serve as a potential diagnostic and prognostic biomarker for colorectal cancer [27]. Many scientists discovered the overexpression of serum exosomal miR-378, miR-7977, and miR-106b in lung cancer, and such upregulation is highly correlated with positive lymph node metastasis and TNM staging, suggesting that these three miRNAs may be promising non-small-cell biomarkers in lung cancer screening and monitoring [28–30]. Another study on the expression of serum exosomal miRNAs in hepatic cancer indicated that the expression of serum exosomal miR-10b-5p increases in hepatic cancer; thus, it could be considered a potential diagnostic biomarker for early hepatic cancer. To assess the diagnostic and prognostic values of serum exosomal miR-10b-5p in hepatic cancer, ROC curve analysis was performed on miR-10b-5p, which presented a sensitivity of 90.7% and a specificity of 70.5% in hepatic cancer diagnosis. It was also found that serum exosomal miR-215-5p acts as a prognostic biomarker for hepatic cancer [31]. Another study revealed that serum exosomal miR-320d could become a noninvasive biomarker in the diagnosis and prognosis of hepatic cancer [32]. In a study using cationic liposome-based nano-biochips, the researchers detected increased plasma exosomal miR-21 and miR-10b in pancreatic cancer patients, suggesting that plasma exosomal miR-21 and miR-10b serve as noninvasive diagnostic biomarkers for early pancreatic cancer [33]. Relying on gene chip technology, another study on pancreatic cancer discovered that miR-196b-3p and miR-204-3p in the serum exosomes of pancreatic cancer patients act as diagnostic biomarkers for pancreatic cancer [34]. A study on thyroid cancer using small RNA sequencing revealed that miR-485-3p and miR-4433a-5p in plasma exosomes are diagnostic biomarkers for papillary thyroid cancer and that miR-485-3p can be used to distinguish high-risk from low-risk papillary thyroid cancer [35]. Over the years, these studies have fully confirmed the role of miRNAs in the exosomes as diagnostic biomarkers for tumors.

In this study, the expression of exosomal miRNAs was compared between healthy subjects and gastric cancer patients to reveal their role in gastric cancer. Serum exosomes were successfully separated and extracted, and the results were consistent with those obtained by several studies on serum exosomes [36, 37]. TEM showed their form of goblet vesicles or irregular circles. NTA exhibited an average particle size of 102.3 nm. Western blot analysis confirmed the characteristics of exosomes and identified the positive expressions of the exosomal biomarkers CD9, CD63, TSG101, and calnexin. These findings provided strong evidence that these vesicles were exosomes. Small RNA deep sequencing was performed on the extracted exosomes, and the serum exosomal miRNA profiles of healthy subjects and gastric cancer patients were comprehensively analyzed using bioinformatics analysis. In the profiles of the complete transcription groups, 15 different types of RNAs were identified, and the attention was focused on miRNAs. The serum exosomal miRNA profiles of the two groups were different, and their miRNAs accounted for 28.01% and 26.26% of the total RNA, respectively. At the same time, the most abundant component was miRNA since the content of rRNA, scRNA, snoRNA, snRNA, and other RNAs was extremely low. A total of 698 known miRNAs in group N and 459 known miRNAs in group T were identified, including 445 shared miRNAs and 267 specific miRNAs. These results suggested the existence of differences between the two groups in serum exosomal miRNA profiles. Group T possessed 3,629 exosomal miRNAs, with 35 significantly dysregulated miRNAs, including 11 upregulated and 24 downregulated ones (Figure 4(b)). Among them, 8 existing human miRNAs, 24 known miRNAs, and 3 novel miRNAs were found. These data suggested that these differentially expressed miRNAs can serve as diagnostic biomarkers for gastric cancer, although it should be further validated through trials.

The target genes of the 8 known differentially expressed miRNA were predicted and selected for GO and KEGG function enrichment analysis (*P* < 0.05). To further reduce errors through the analysis, *P* < 0.05 after correction was adopted as the screening condition, cancer-related pathways were selected for functional enrichment analysis, and some pathways were discovered as having a close correlation with tumors. For instance, the MAPK and Rap1 signaling pathways are important pathways affecting tumor invasion and metastasis. Wnt signaling pathway and apoptosis are key pathways influencing tumor proliferation and apoptosis. As a result of the upregulation or downregulation of miRNAs in the exosomes, changes may occur in the target genes of the above signaling pathways. The 8 differentially expressed miRNAs participated in multiple cancer-related pathways, suggesting that these miRNAs play essential roles in cancer. This analysis proposed a solid foundation for further studies on the role of serum exosomes in gastric cancer. KEGG pathway enrichment analysis also revealed interactions and biological functions of target genes and classified all the enrichment pathways into six subgroups, such as metabolism, genetic information processing, environmental information processing, cellular process, organism system, and human diseases. The miRNAs investigated in this study played vital roles in the information processing, transduction, and tumor diseases, which exactly corresponded to the close correlation of exosomes with communication and tumors. The subgroup ranking in the first place was the environmental information processing subgroup (mainly signal transduction and interactions with signal molecules), followed by the subgroup of human diseases (mainly tumors and infectious diseases, especially tumor diseases). These observations confirmed the transmission role of the miRNAs encapsulated in the exosomes in biological processes and their close correlation with tumors, further providing some references for investigating the function of serum exosomes and the biological processes in which they participated. GO and KEGG analyses revealed the close correlation of these miRNAs with tumor proliferation, invasion, metastasis, apoptosis, and other related pathways, indicating the direction of the follow-up research. Therefore, specific trials should be performed in future studies to validate the targets and pathways regulated by these differentially expressed miRNAs.

Four downregulated miRNAs from these differentially expressed miRNAs were selected. The results showed that the expression of miR-10401-3p, miR-1255b-5p, and miR-6736-5p in the serum exosomes from gastric cancer patients were significantly lower than those in the serum exosomes from healthy subjects, while no statistically significant difference in the expression of miR-8072 was found between the two groups. In addition, the 3 miRNAs showing a statistically significant difference between the two groups exhibited a high accuracy in gastric cancer diagnosis. All these findings demonstrated the great potential of miR-10401-3p, miR-1255b-5p, and miR-6736-5p in serum exosomes as diagnostic biomarkers for gastric cancer. However, the serum exosomal miRNA sequencing performed by Wang et al. [38] in gastric cancer revealed that miR-19b-3p and miR-106a-5p in the serum exosomes act as novel potential diagnostic biomarkers for gastric cancer. Tang et al. [39] performed serum exosomal miRNA sequencing in early gastric cancer and discovered that miR-92b-3p, miR-146b-5p, miR-9-5p, and let-7g-5p in serum exosomes are noninvasive biomarkers in early gastric cancer diagnosis. Zhang et al. [40] performed plasma exosomal miRNA sequencing in gastric cancer, reporting that miR-10b-5p, miR-101-3p, and miR-143-5p are candidate biomarkers in the lymphatic metastasis, ovarian metastasis, or hepatic metastasis of gastric cancer. According to these findings, they classified gastric cancer patients according to their metastasis types. These studies confirmed the role of exosomal miRNAs as diagnostic biomarkers for gastric cancer. However, the sequencing studies mentioned above identified specific diagnostic biomarkers different from those identified in this study, potentially because of differences in sample source, sample volume, exosome separation, extraction method, trail design, analysis method, grouping, sequencing platform, or data processing method.

This study had some limitations. First, the sample size was small since the serum exosomes from three normal subjects and six gastric cancer patients (three in early stage and three in advanced stage) were considered; a problem of poor homogeneity of the samples was also found. As a result, only a few differentially expressed miRNAs were screened, although they were sufficient to prove the presence of differences between healthy subjects and gastric cancer patients in serum exosomal miRNA profiles. Second, given the small number of early gastric cancer patients and the insufficiency of early-stage samples for confirmation by qRT-PCR, early-stage and advanced-stage samples were combined to form group T. The problem with this approach was that the screened miRNAs could only be used to screen diagnostic biomarkers for gastric cancer, without distinguishing early-stage from advanced-stage gastric cancer or establish associations with clinical characteristics.

In conclusion, the differential expression profiles of serum exosomal miRNAs from healthy subjects and gastric cancer patients were investigated using small RNA sequencing, and some existing, known, and novel miRNAs were discovered. Although the novel miRNAs still need further research, 3 existing miRNAs might serve as noninvasive, novel diagnostic biomarkers for gastric cancer and as targets for gastric cancer treatment. Thus, this study provided new insights into the expression profiles and distribution characteristics of exosomal miRNAs in gastric cancer, which could be used for further exploration of the role of intercellular communication through exosome-mediated miRNAs in gastric cancer. However, the results of this study still need to be further validated by more multicenter, retrospective studies.

## 10. Prospect

Currently, exosomes are a research hotspot, and their role in many fields, especially in tumor treatment, is being gradually recognized. As noninvasive biomarkers, they can be conveniently and accurately used in the screening, diagnosis, and treatment of cancer and assessing the efficacy of a specific therapy. They can also be used in combination with current diagnostic indices and data on the expression of serum exosomal miRNAs to improve the accuracy of early gastric cancer diagnosis. Considering that exosomes carry and transfer miRNAs and transmit information among tumor cells, a potentially effective approach might be to focus on stopping the initiation and progression of tumors by blocking the channels of transfer, thus offering a new strategy for tumor treatment.

## Figures and Tables

**Table 1:** Primer sequences of U6 and four miRNAs for qRT-PCR.

U6/miRNA	Forward primer sequences	Reverse primer sequences
U6	GGAACGATACAGAGAAGATTAGC	TGGAACGCTTCACGAATTTGCG
miR-10401-3p	AAAATCCCGCCCGCCGAA	mRQ3′primer provided with miR-X miRNA first-stand synthesis kit (Takara)
miR-1255b-5p	GGATGAGCAAAGAAAGTGGTTAAA	
miR-6736-5p	GGTGAGGGCATCTGTGGTAAA	
miR-8072	ATCGGGGAGGTAGGCAGAAA	

**Table 2:** List of significantly dysregulated 8 miRNAs in group N vs. group T.

	miRNA	TPM (N) mean	TPM (T) mean	log2 (fc)	Regulation	*P*-value
N vs T	miR-642a-3p	0.9369	13.27	3.82	Up	0.0181
	miR-1228-3p	3.55	0.01	−8.47	Down	0.0184
	miR-1255b-5p	4.93	0.01	−8.94	Down	0.0096
	miR-1304-5p	4.18	0.01	−8.65	Down	0.0275
	miR-10401-3p	76.95	29.55	−1.38	Down	0.0487
	miR-6736-5p	3.28	0.01	−8.36	Down	0.0100
	miR-6747-3p	3.83	0.01	−8.58	Down	0.0317
	miR-8072	3.06	0.01	−8.25	Down	0.0339

**Table 3:** Number of predicted gene targets for the selected eight miRNAs of biological interest.

miRNA	Total targets predicted (N)	Targets with score <−35 energy (kcal/mol) (N)
miR-10401-3p	964	66
miR-6736-5p	2408	26
miR-1255b-5p	3447	1
miR-642a-3p	3238	0
miR-1228-3p	2558	19
miR-1304-5p	3486	8
miR-8072	1394	347
miR-6747-3p	3761	54

**Table 4:** KEGG and GO pathway enrichment analysis on cancer-related pathways in which target genes participated.

Id	Num	*P* value	*Q* value	Per	Ratio
Pathways in cancer	662	3.38*E* − 05	3.17*E* − 04	9.761	0.244
MAPK signaling pathway	402	0.03151	9.75*E* − 02	5.927	0.232
Proteoglycans in cancer	323	1.89*E* − 09	1.24*E* − 07	4.763	0.287
Ras signaling pathway	322	1.10*E* − 06	1.90*E* − 05	4.748	0.271
cAMP signaling pathway	294	6.27*E* − 06	7.62*E* − 05	4.335	0.268
Rap1 signaling pathway	274	0.000354	2.15*E* − 03	4.04	0.257
Oxytocin signaling pathway	253	1.02*E* − 06	1.85*E* − 05	3.73	0.28
Wnt signaling pathway	231	1.82*E* − 06	5.36*E* − 05	3.406	0.279
Hepatocellular carcinoma	226	0.000364	2.17*E* − 03	3.332	0.262
cGMP-PKG signaling pathway	222	0.005342	2.14*E* − 02	3.273	0.249
Apoptosis	209	0.002303	1.07*E* − 02	3.082	0.255
FoxO signaling pathway	199	0.000917	4.77*E* − 03	2.934	0.261
mTOR signaling pathway	196	0.000353	2.15*E* − 03	2.89	0.266
ErbB signaling pathway	185	4.65*E* − 08	1.52*E* − 06	2.728	0.306
Breast cancer	174	0.000154	1.07*E* − 03	2.566	0.274
Colorectal cancer	166	0.002736	1.23*E* − 02	2.448	0.26
TNF signaling pathway	154	0.02415	8.06*E* − 02	2.271	0.247
Pancreatic cancer	150	0.002697	1.23*E* − 02	2.212	0.263
Cell adhesion molecules (CAMs)	136	0.002295	3.53*E* − 01	2.005	0.231
NF-kappa B signaling pathway	92	0.003521	7.71*E* − 01	1.357	0.217

**Table 5:** Correlation of serum exosomal miR-10401-3p, miR-1255-5p, and miR-6736-5p expression (ΔCt) and clinicopathological features of patients with GC

Features	Number	miR-10401-3p		miR-1255-5p		miR-6736-5p	
		Mean ± SD	*P* value	Mean ± SD	*P* value	Mean ± SD	*P* value
Age (years)							
≥60	12	1.04 **±** **1.02**	0.70	0.07 **±** **0.05**	0.755	0.32 **±** **0.18**	0.727
<60	12	0.87 **±** **1.05**		0.06 **±** **0.05**		0.35 **±** **0.29**	
Gender							
Male	14	0.86 **±** **1.01**	0.591	0.058 **±** **0.045**	0.643	0.35 **±** **0.22**	0.748
Female	10	1.09 **±** **1.06**		0.068 **±** **0.05**		0.31 **±** **0.27**	
Tumor size							
5 cm	11	1.49 **±** **1.25**	0.029	0.59 **±** **0.51**	0.766	0.38 **±** **0.27**	0.309
<5 cm	13	0.51 **±** **0.44**		0.65 **±** **0.49**		0.28 **±** **0.20**	
Tumor differentiation							
Well	5	0.52 **±** **0.56**	0.288	0.49 **±** **1.123**	0.526	0.24 **±** **0.20**	0.330
Moderate + poor	19	1.07 **±** **1.09**		0.07 **±** **0.05**		0.36 **±** **0.24**	
TNM stage							
I–II	6	0.73 **±** **0.48**	0.533	0.05 **±** **0.02**	0.369	0.15 **±** **0.18**	0.03
III–IV	18	1.03 **±** **1.14**		0.07 **±** **0.05**		0.39 **±** **0.22**	
Lymphatic node metastasis							
Negative	15	0.93 **±** **0.96**	0.855	0.04 **±** **0.02**	0.004	0.26 **±** **0.18**	0.045
Positive	9	1.01 **±** **1.17**		0.11 **±** **0.05**		0.46 **±** **0.28**	

## Data Availability

The data used to support the findings of this study are included within the article.

## Additional Points

*Summary.* This study analyzed the expression of serum exosomal miRNAs from healthy subjects and gastric cancer patients using small RNA sequencing. The results of the two groups were compared by the bioinformatics analysis to evaluate the statistical difference. Quantitative reverse transcription-polymerase chain reaction (qRT-PCR) was used to confirm the results of the selected differentially expressed miRNAs that could be thus considered biomarkers with potential diagnostic values.
